# Phenol as a Tethering Group to Gold Surfaces: Stark Response and Comparison to Benzenethiol

**DOI:** 10.1021/acs.jpclett.3c02058

**Published:** 2023-09-13

**Authors:** Sevan Menachekanian, Carlos Mora Perez, Anuj K. Pennathur, Mattew J. Voegtle, Drew Blauth, Oleg V. Prezhdo, Jahan M. Dawlaty

**Affiliations:** † Department of Chemistry, University of Southern California, Los Angeles, California 90089, United States; ‡ Theoretical Physics and Chemistry of Materials, Los Alamos National Laboratory, Los Alamos, New Mexico 87545, United States; § Center for Nonlinear Studies, Los Alamos National Laboratory, Los Alamos, New Mexico 87545, United States; ∥ Department of Chemistry, University of Colorado Boulder, Boulder, Colorado 80309, United States

## Abstract

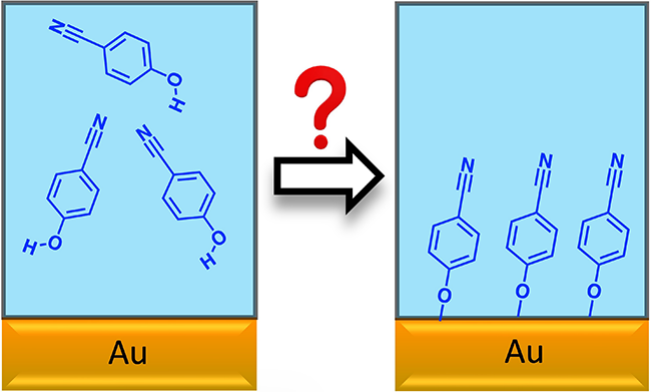

Understanding the adsorption of organic molecules on metals is important in numerous areas of surface science, including electrocatalysis, electrosynthesis, and biosensing. While thiols are commonly used to tether organic molecules on metals, it is desirable to broaden the range of anchoring groups. In this study, we use a combined spectroelectrochemical and computational approach to demonstrate the adsorption of 4-cyanophenols (CPs) on polycrystalline gold. Using the nitrile stretching vibration as a marker, we confirm the adsorption of CP on the gold electrode and compare our results with those obtained for the thiol counterpart, 4-mercaptobenzonitirle (MBN). Our results reveal that CP adsorbs on the gold electrode via the OH linker, as evidenced by the similarity in the direction and magnitude of the nitrite Stark shifts for CP and MBN. This finding paves the way for exploring new approaches to modify electrode surfaces for controlled reactivity. Furthermore, it highlights adsorption on metals as an important step in the electroreactivity of phenols.

Self-assembled monolayers (SAMs) are molecular assemblies with functional groups that bind to solid surfaces such as gold, silver, and copper.^1^ Monolayers with thiol functional groups are among the most studied SAMs.^2−4^ Thiol SAMs have a wide range of applications in industry, including in electronic devices,^5^ biosensors,^6−10^ microfabrication,^5,11^ batteries,^12^ anticorrosion coatings for metals,^13−15^ drug delivery,^16^ and catalysis.^17^ Additionally, thiol SAMs can be used on electrode surfaces to study interfacial processes and reactions. Hildebrandt and co-workers have used thiol monolayers to study protein structure and dynamics.^18−20^ Mayer and co-workers have employed aromatic SAMs 4-mercaptobenzoic acid and 4-mercaptobenzonitrile (MBN) to study acid–base interfacial equilibria.^21^ Some of us have employed thiol-based SAMs to study the interfacial electric field, ionic structures near the electrodes, and the formation of interfacial adducts.^22−27^

The natural analog of aromatic thiol is phenol, a very common functional group in a wide range of organic and biological molecules. Hence, it is desirable to explore the surface adsorption of phenols on metal surfaces and compare the results to those of their thiol counterparts. While the interaction of aromatic thiols with metal surfaces has been widely studied, the adsorption of phenols on these surfaces has received little attention. Moreover, the orientation of phenols upon adsorption on metal surfaces remains a topic of debate. One study showed that phenols and 4-cyanophenols in a basic medium adsorb onto the gold surface in a perpendicular orientation via the oxygen atom.^28,29^ Reflectance and capacitance measurements have shown that phenols adsorb to the gold surface at negative potentials via the π-interaction. However, they adopt a perpendicular configuration at positive potentials by forming a strong covalent bond between the oxygen atom and gold.^30^ Another research investigated the adsorption of phenols on gold in an aqueous medium. However, the study did not provide information on the phenols’ adsorption geometry.^31^ A separate study demonstrated that phenols can adopt different adsorption geometries on metal surfaces depending on their concentration. Specifically, the study found that at low concentrations, phenols adsorb onto gold in a flat orientation, while at higher concentrations, they exhibit a more upright configuration.^32^ The adsorption of phenols and their geometry with respect to gold surfaces remain a topic of ongoing debate, with inconclusive reports. Hence, further investigation in this area is necessary. Specifically, it is crucial to compare phenols’ adsorption behavior with that of thiols to understand whether phenols adsorb and behave similarly.

In this work, we use vibrational spectroscopy to show the adsorption of CP on gold. Our technical and experimental approaches differ from previous work in two key aspects. First, we use surface-enhanced Raman spectroscopy (SERS) by measuring the nitrile vibration as a marker to confirm the adsorption of CP on the gold electrode. Second, we measure the vibrational frequency of nitrile as a function of potential over a relatively wide range (+0.6 to −0.8 V vs Ag/AgCl). The observed frequency shifts due to potential are similar to that of MBN, suggesting that the adsorbed CP and MBN molecules have similar bonding structures to the substrate. Finally, to ensure that the binding is stable and that molecules are not physisorbed, we have subjected the electrodes after adsorption to sonication before all of our spectroelectrochemical measurements.

The frequency shift of molecules in response to electric fields is more generally known as the Stark shift. Vibrational Stark shift has been used to infer the electrostatics of biomolecules and enzymes.^33−35^ More specifically, the frequency change of adsorbed molecules in response to electrochemical potential is a powerful tool for understanding the electrostatics and solvation environment of the interface.^36^ A molecule may respond to the potential if it is adsorbed to the surface or is within the electric double layer and can feel the polarization from the electrode. Our observation of the CP molecule’s Stark response and its similarity to MBN confirm that CP is adsorbed to the surface with the oxygen atom. Additionally, computational approaches are utilized to estimate the adsorption energies of Au–O and Au–S bonds and determine the adsorption geometries for both surface-bound species.

First, we compare the Raman spectra of CP and MBN in bulk and adsorbed on the electrode. Figure 1a displays the Raman spectra of bulk CP and MBN in ethanol obtained under similar experimental conditions. The Raman signals for both molecules are comparable, indicating that the nitrile stretching mode has a similar Raman cross section for both molecules. The small difference in frequency between the two is ascribable to the differences in the electron-withdrawing strengths (Hammett parameter σ_p_) between SH and OH,^37,38^ as well as their relative response to the hydrogen-bonding solvent. Note that this difference is not the central point of this work and will not be discussed further.

**Figure 1:**
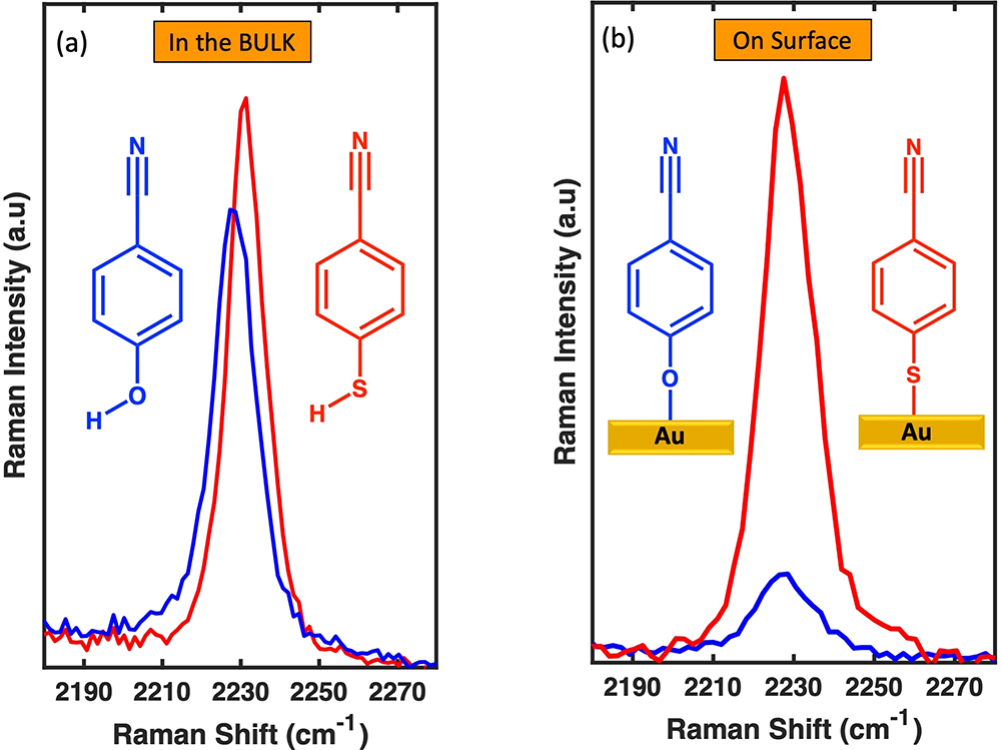
(a) Raman spectra of 0.1 M CP and 0.1 M MBN in ethanol obtained under similar experimental conditions, revealing that their Raman cross sections are comparable. (b) SERS spectra of CP and MBN on gold, indicating the successful adsorption of CP albeit with lower coverage.

Figure 1b shows the SERS spectra of CP and MBN, prepared from a solution of similar concentration and equal soaking time (48 h). The spectra are obtained in a 0.1 M NaClO_4_ solution. The SERS signal of MBN exhibits a significant 6-fold increase compared to CP. Given that the nitrile Raman cross section for CP and MBN is nearly the same in the bulk solution, it is reasonable to attribute the difference to surface coverage. This disparity in surface coverage can be attributed to the lower adsorption energy and slower adsorption kinetics of CP relative to those of MBN, as discussed later.

Figure 2 depicts the change in the nitrile stretching frequency of CP and MBN as a function of the electrode potential in 0.1 M NaClO_4_. Each frequency value represents the average of three independent measurements on different samples recorded after applying potentials with a 0.1 V increment. All frequency values are referenced with respect to the frequency at 0 V vs Ag/AgCl.

**Figure 2:**
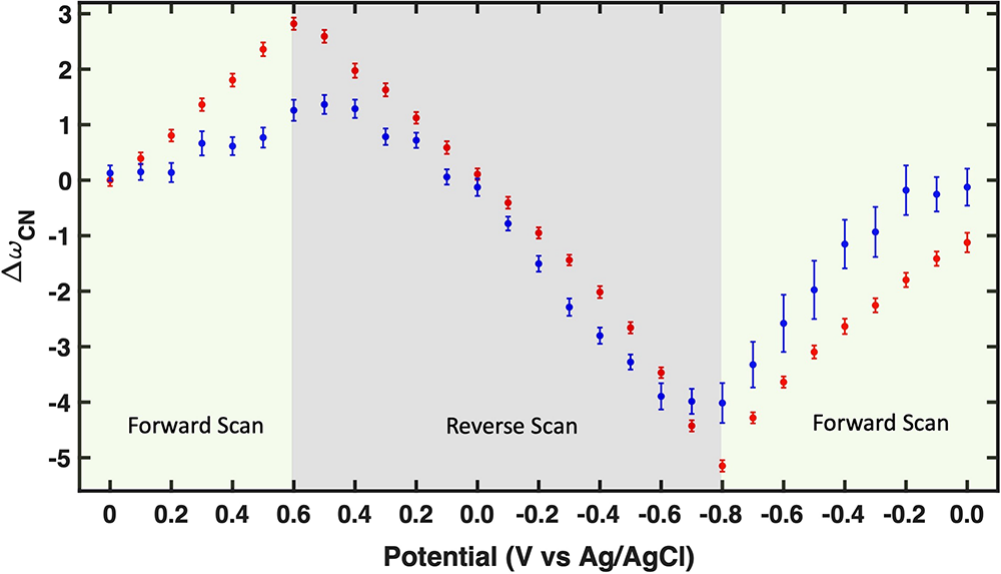
Frequency shift of MBN (red) and CP (blue) as a function of applied electrochemical potential relative to 0 V vs Ag/AgCl. The electrolyte is 0.1 M NaClO_4_ in water. The electrode potential begins at 0 V and is swept forward to positive potentials, followed by a reverse scan to negative values and then back to 0 V. Each frequency value represents the average of three independent measurements.

The behavior of CP is qualitatively very similar to that of MBN. The potential response of CP seems to deviate slightly in the forward scan and lags behind that of MBN by about 2 cm^–1^. However, in the range of 0.4 V to −0.6 V, the behavior is very similar. Note that each trace is the average of three independent measurements on three samples.

The potential-induced Stark shifts of the nitrile stretching mode for MBN have been reported in the literature^23,37,39,40^ in the range of 5 to 8 cm^–1^/V. In this work, a Stark tuning rate of 5.35 cm^–1^/V was observed in the potential range of −0.4 to 0.6 V, consistent with previous reports. Note that the Stark tuning rate is influenced by several factors, including ionic strength and^23^ electrolyte identity.^37^

Interestingly, the Stark tuning rate for CP is 5.34 cm^–1^/V in this range, which is very similar to that of MBN. This similarity indicates that MBN and CP molecules have the nitrile group located approximately at the same distance from the electrode and are covalently bound to the gold electrode via the sulfur and oxygen atoms, respectively.

It is very unlikely that CP molecules attach to gold via the nitrogen of the nitrile group. Recently, it has been demonstrated that bond formation between a nitrile group and metal ions results in a significant blue shift in the nitrile frequency.^41^ If CP were adsorbed onto the gold surface via the nitrile nitrogen, this would cause a significant blue shift in the nitrite frequency and likely an inverse Stark shift.

We calculated the vibrational frequency of the nitrile group for both molecules as a function of potential. This was achieved by varying the charge of the metal slab from −1 to +1, as outlined in the Computational Methods section. It is worth noting that in using the PBE functional, the absolute frequency values tend to be overestimated. Consequently, the relative trends are of primary significance and carry relevant information. As shown in Figure 3, the nitrile frequencies of MBN consistently exhibit higher values than those of CP, irrespective of the charge levels. Translating charge into an equivalent electrochemical potential for better comparison with experimental values presents a significant challenge. This process often necessitates the implementation of sophisticated methodologies, higher computational costs, and in certain instances an explicit account of the electrolyte.^42^ Given that the exact comparison between the calculated and experimental values is not the central focus of our current investigation, we have chosen to confine our discussion to a comparative analysis of the trends of CP and MBN.

**Figure 3:**
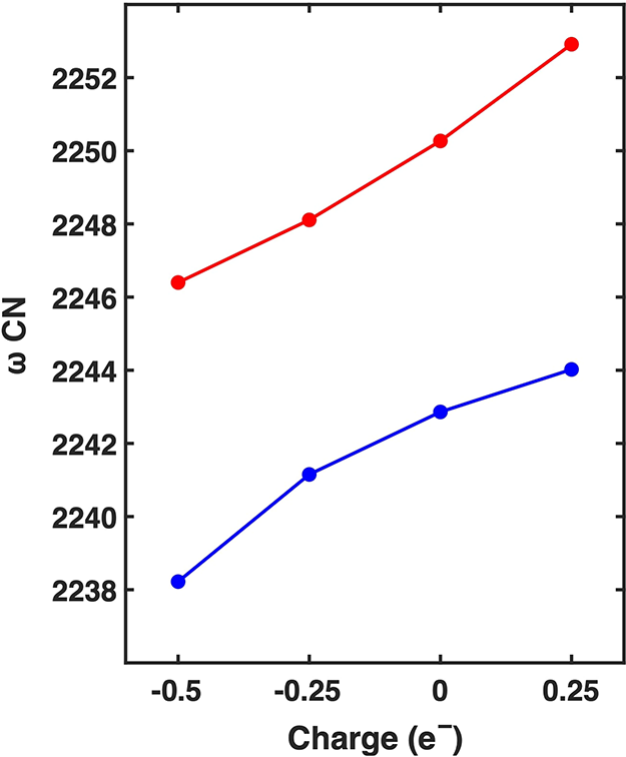
Calculated nitrile stretching frequencies for MBN (red) and CP (blue) adsorbed on a slab of gold at different total net charges for the system. Consistent with the experimental results, the nitrile frequency for both molecules shows a blue shift with a more positive charge on the gold slab.

In agreement with experimental findings, introducing a positive charge to the system (similar to applying a positive potential to the electrode) results in an increased nitrile frequency for both MBN and CP, as illustrated in Figure 3. The slightly nonlinear behavior of the frequency in response to charge is consistent with previous studies.^36,37^ Detailed information on the calculated nitrile vibrational frequency across the charge range of −1 to +1 can be found in the Supporting Information.

Next, we investigate the kinetics of CP adsorption and compare the results to MBN. The adsorption kinetics of the Au–S bond have been thoroughly investigated in the literature. The typical adsorption saturation time corresponding to maximum coverage for thiol derivatives on gold can range from seconds^43,44^ to minutes,^45−47^ and in some cases, it may even take several hours^48^ for the adsorbed monolayers to relax and rearrange fully.

Figure 4 illustrates the peak Raman intensity as a function of time. Our experiments suggested that MBN is most ideally adsorbed from a 1 mM solution in ethanol. To the best of our knowledge, no existing literature has reported the adsorption kinetics of phenol on gold. The figure shows that the adsorption kinetics for MBN is significantly faster than CP’s. After approximately 20 min, the Raman intensity for MBN reaches a plateau, indicating adsorption saturation consistent with the reported literature.^45,46^ The signal for CP is about 10 times smaller initially and rises much slower. Even after 40 min, it did not show a plateau. The difference in adsorption kinetics between CP and MBN may be attributable to the higher adsorption energy of Au–S compared to Au–O shown by our computational work below.

**Figure 4:**
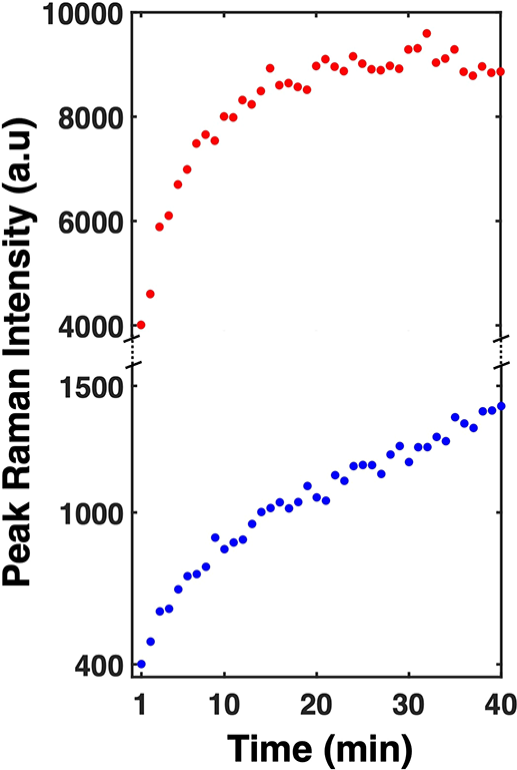
Time-dependent peak Raman intensity for 1 mM MBN (red) and 1 mM CP in ethanol (blue).

Figure 5 shows the binding energies for MBN and CP at various charges. At zero charge, the Au–S binding energy is −2.23 eV (−53.7 kcal/mol), which is in good agreement with the reported value in the literature.^49,50^ The calculated binding energy for CP is −0.33 eV (−7.6 kcal/mol). Both molecules demonstrate a linear relationship between binding energy and adding charge to the gold slab. However, MBN displays a slope (−0.33 eV/charge) that is more negative than that of CP (−0.062 eV/charge). Noticeably, at +1 charge, the difference in binding energy is the highest. These disparities may arise from the larger polarizability of the gold–sulfur bond compared to that of the gold–oxygen bond. The more covalent nature of the gold–sulfur bond makes it more susceptible to electrode polarization.

**Figure 5:**
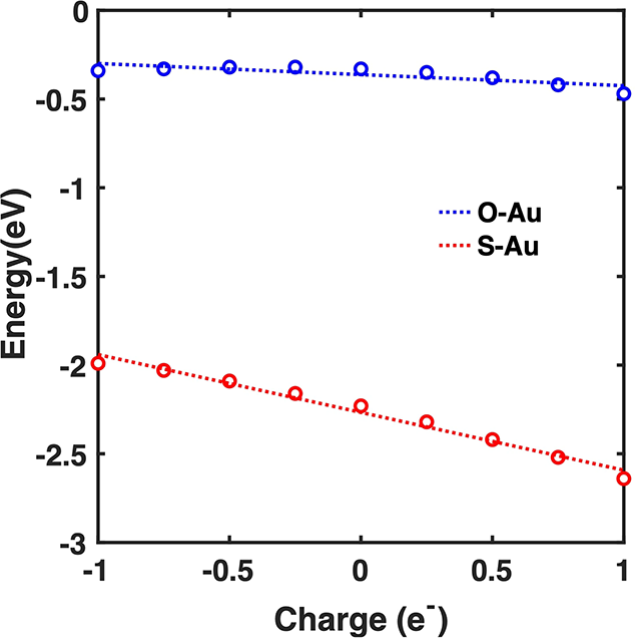
Binding energy of CP (blue) and MBN (red) shows a linear dependence with increasing charge. MBN has stronger binding to the gold surface and higher sensitivity to charge with a slope of −0.33 eV/charge, while CP has a slope of −0.062 eV/charge.

Additionally, previous studies have shown that 4-cyanophenols form an ordered structure on the Au(111) surface, as expected for similar phenol structures on an Au(111) surface.^51^ The structural order is consistent with calculated optimized geometries utilized in this study, as shown by calculated Au–O bond lengths and CP–Au angles shown in the Supporting Information. CP has a more perpendicular angle with respect to the surface at 89°, whereas MBN has a smaller angle to the surface 85° regardless of the charge in the system, consistent with structural ordering previously observed.

Next, we compare the electron densities at the interfacial regions and across the molecule. Figure 6 shows the charge density differences between free and adsorbed molecules for MBN and CP for a neutral gold slab calculated based on eq 2. The colors red and blue correspond to volumes that gain and lose electron density, respectively. The two molecules exhibit qualitatively similar behavior. A slightly higher density of electrons is observed near the Au–S bond compared with the Au–O system, indicating a stronger affinity of sulfur to gold.

**Figure 6:**
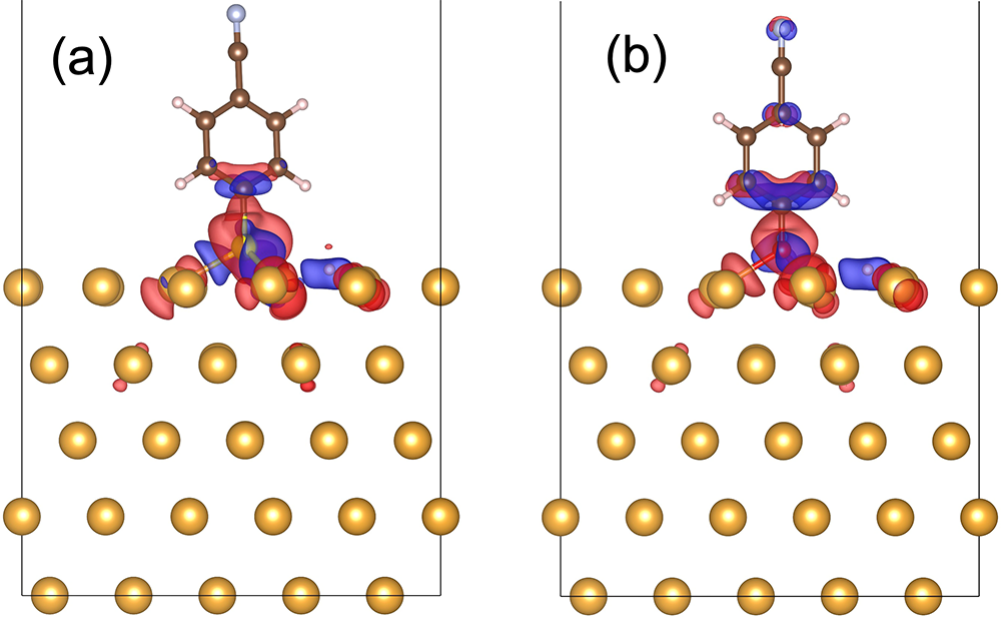
Charge density difference plots of MBN (a) and CP (b) calculated according to eq 2. The negative and positive charge build-up is shown by red and blue colors, respectively, with an isovaule of 0.0025 e/bohr^3^. The two molecules show qualitatively similar charge build-up upon adsorption. However, the charge build-up for MBN in the gold thiol bond region is slightly more pronounced.

In conclusion, we investigated the adsorption of CP onto a gold surface and compared it to MBN. Our experimental and computational results suggest that CP can adsorb to the gold surface through the oxygen linker but with a lower surface coverage and slower kinetics compared to those of MBN. Our findings are relevant to electrocatalysis and electroadsorption studies of molecules containing phenol derivatives. Phenols, catechols, and polyphenols are molecules that have relevance to organic chemistry, pharmaceuticals, biology, and environment. We suspect that adsorption of such compounds on metal surfaces may also show analogous behavior. Therefore, the initial steps of the reactivity of such compounds in the presence of metals may occur via adsorption by the oxygen linkers. In particular, adsorption of catechols on metals is of interest because the two adjacent oxygens may provide enhanced surface adsorption and perhaps larger Stark sensitivity due to more direct electronic coupling with the surface. Furthermore, since thiols also adsorb on silver, copper, and some other metals, it is likely that phenols may also exhibit similar behavior on these metals, which we plan to explore in the future.

## Experimental Methods

We employed a procedure for electrode preparation and spectroelectrochemical measurements described in our previous work.^26^ In brief, an SERS substrate was prepared by heating and flattening a 1 mm thick gold wire from Surepure Chemetals Inc. The gold substrate was then sanded with sandpaper until the surface became shiny, followed by sonication in ethanol for 10 min. Subsequently, a thin layer of gold was deposited on the substrate using a gold electrodeposition solution (Gold Touch Inc.). The gold wire was further sonicated in ethanol for 10 min. Afterward, it was immersed in a sealed container filled with a 10 mM solution of each chemical in ethanol for 48 h to allow for monolayer adsorption. The gold surface was then rinsed and sonicated for 2 min in ethanol. Because of the lower surface coverage of CP compared to MBN, it is necessary to follow the electrode preparation procedure meticulously to obtain good SERS signal. We also noted that depositing CP on the surface from a basic aqueous solution produced good results, perhaps suggesting that deprotonation of CP may be a necessary first step. We observe that the CP monolayers may be as stable as the MBN monolayers in dry condition. However, under aqueous conditions and pH variations, they may come off more readily. This will be the subject of our future experimental work.

A home-built cell made of Teflon was used for spectroelectrochemical measurements. SERS data were obtained using a HORIBA XploRA micro-Raman microscope with a 785 nm excitation source. The typical accumulation time for data collection was around 20 s. Electrochemical measurements were performed by using a Ag/AgCl (3 M KCl) reference electrode, a gold counter electrode, and a Gamry potentiostat (model 1010 B). All chemicals were purchased from Sigma-Aldrich without further purification.

## Computational Methods

(DFT)^52−54^ ab initio molecular dynamics (AIMD) with the projector-augmented-wave (PAW) potentials^55,56^ and the Perdew–Burke–Ernzehof (PBE) functional under the generalized gradient approximation^57,58^ was employed for all systems. The electronic structure calculations were performed within the Vienna ab initio simulation package (VASP).^59−62^ The PAW PBE versions included in the POTCAR files for each species were PAW-PBE Au 04October2007, PAW-PBE H 15June2001, PAW-PBE C 08Apr2002, PAW-PBE O 08Apr2002, PAW-PBE N 08Apr2002, and PAW-PBE S 06Sep2000. The Au slab was built with a 111 exposed surface with a 5-layer thickness and 5 × 5 in the *x*–*y* plane. During the VASP calculations, we utilized a sizable plane-wave basis energy cutoff (ENCUT) of 520 eV, a 1 × 1 × 1 Γ centered k-point mesh to sample the Brillouin zone. Studies of molecules adsorption on Au(111) show the need for van der Waals (vdW) dispersion interactions.^63^ Thus, we implemented the Tkatchenko–Scheffler (TS) method with iterative Hirshfeld partitioning for all the simulations.^64−67^ Spin-polarized calculations were omitted since previous studies have demonstrated their negligible impact on the reported results regarding adsorption on Au(111) surfaces.^68^ Given the large size of the unit cell, only the γ point was used. This slab was constructed based on the bulk lattice constant of 4.17 Å, as determined from previous calculations on cubic Au bulk crystal, data retrieved from the Materials Project for Au (mp-81) from database version v2022.10.28.^69,70^ The bottom two layers of the Au slab were frozen to the bulk geometry in all calculations. The unit cell height was also set to 39.63 Å, to provide a 30 Å vacuum between Au slab layers in the *z*-direction. The molecules were placed above the top Au layer in an fcc chemisorption site shown to be a favorable position in previous studies for thiols and oxygen on Au(111).^68,71−73^ For consistency, the oxygen molecule was also placed in the same position before optimization. Additionally, CP and MBN were spaced such that O–O and S–S have a bond distance of 14.7 Å, sufficient to avoid molecule–molecule self-interactions caused by the boundary conditions of the simulations. The harmonic nitrile frequencies are computed by taking the second-order derivatives of the total energy with respect to the ions’ positions using a finite differences approach.^74,75^ In order to reduce the computational cost, all-atom positions were fixed, except for the C and N atoms in the nitrile probe, which were allowed to be displaced. The absorption energy was calculated as the difference in energy between the molecule on the Au surface *E*_Molecule on Au_^(*n*)^ and the sum of the individual components (charged Au slab *E*_Au_^Charged^ and the gas phase molecule *E*_Molecule_^Neutral^):
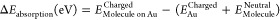
1while the charge density shown is calculated as the difference between the density of the molecule on the Au surface ρ_Molecule on Au_^(charged)^, and the sum of the individual components (charged Au slab ρ_Au_^Charged^, and the gas phase molecule ρ_Molecule_^Neutral^):
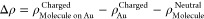
2

## Supporting Information Available

Input files, geometry optimization data, and SERS and Raman spectra of chemicals (PDF)
