# Supplementary material for: Phenol as a Tethering Group to Gold Surfaces: Stark Response and Comparison to Benzenethiol
Source: J Phys Chem Lett. 2023 Sep 13;14(37):8353–9. doi: 10.1021/acs.jpclett.3c02058 (PMC10518863; doi:10.1021/acs.jpclett.3c02058)
Supplement: Supplementary file 1 — jz3c02058_si_001.pdf [file jz3c02058_si_001.pdf]

Supporting Information: Phenol as a Tethering Group to Gold Surfaces: Stark Response and Comparison to Benzenethiol

Sevan Menachekanian,† Carlos Mora Perez,‡ Anuj K. Pennathur,† Mattew J. Voegtle,† Drew Blauth,¶ Oleg V. Prezhdo,*,† and Jahan M. Dawlaty*,†

†*Department of Chemistry, University of Southern California, Los Angeles, California, 90089, USA*

‡*Department of Chemistry, University of Southern California, Los Angeles, California, 90089, USA*

Theoretical Physics and Chemistry of Materials, Los Alamos National Laboratory, Los Alamos, New Mexico 87545, USA

Center for Nonlinear Studies, Los Alamos National Laboratory, Los Alamos, New Mexico 87545, USA

¶*Department of Chemistry, University of Colorado Boulder, Boulder,Colorado 80309, USA*

E-mail: prezhdo@usc.edu; dawlaty@usc.edu

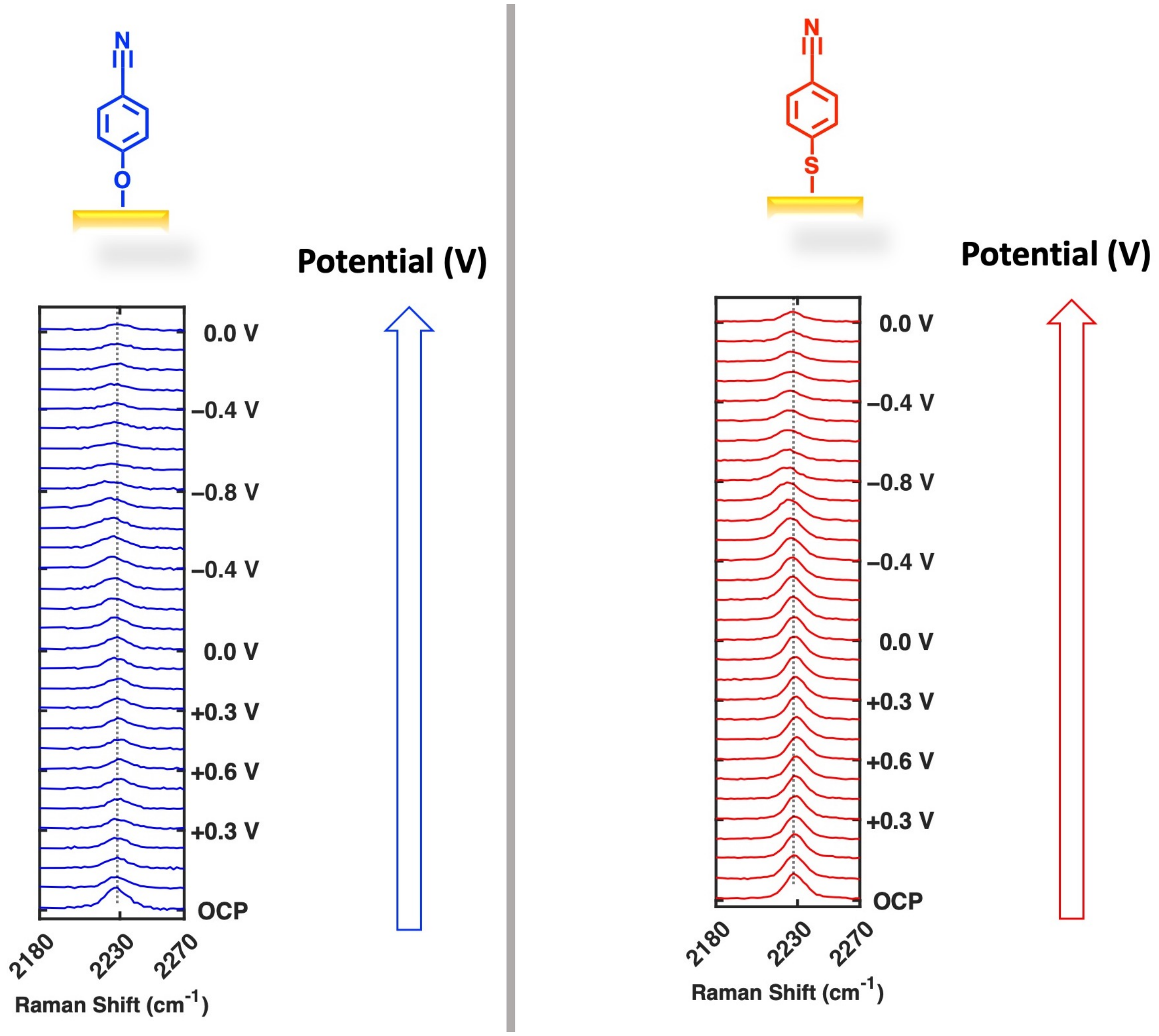

Figure 1: Potential dependent SERS data for the nitrile stretching mode for adsorbed CP on gold (left), and for adsorbed MBN (right) .

Table 1: Binding energy (eV) of CP (red) and MBN (blue) on an Au (111) surface at various charges (0, ±0.25, ±0.5, ±1 electron add/subtracted to the system).

Binding energy (eV)	-1	-0.75	-0.5	-0.25	0	0.25	0.5	0.75	1
S	-1.99	-2.03	-2.09	-2.16	-2.23	-2.32	-2.42	-2.52	-2.64
O	-0.34	-0.33	-0.32	-0.32	-0.33	-0.35	-0.38	-0.42	-0.47

Table 2: Nitrile bond length upon addiction of charge in the system.

C-N Bond	-1	-0.75	-0.5	-0.25	0	0.25	0.5	0.75	1
S	1.16999	1.16896	1.16924	1.1691	1.16896	1.16859	1.16858	1.16855	1.16842
O	1.17097	1.17045	1.17026	1.16994	1.16968	1.16947	1.16953	1.16929	1.16937

Table 3: Nitrile frequency values at various charges from -1 to +1.

Freq	-1	-0.75	-0.5	-0.25	0	0.25	0.5	0.75	1
S	2239.035	2241.456	2246.398	2248.109	2250.268	2252.912	2253.608	2254.022	2255.072
O	2232.21	2236.169	2238.221	2241.151	2242.86	2244.022	2242.72	2243.501	2241.407

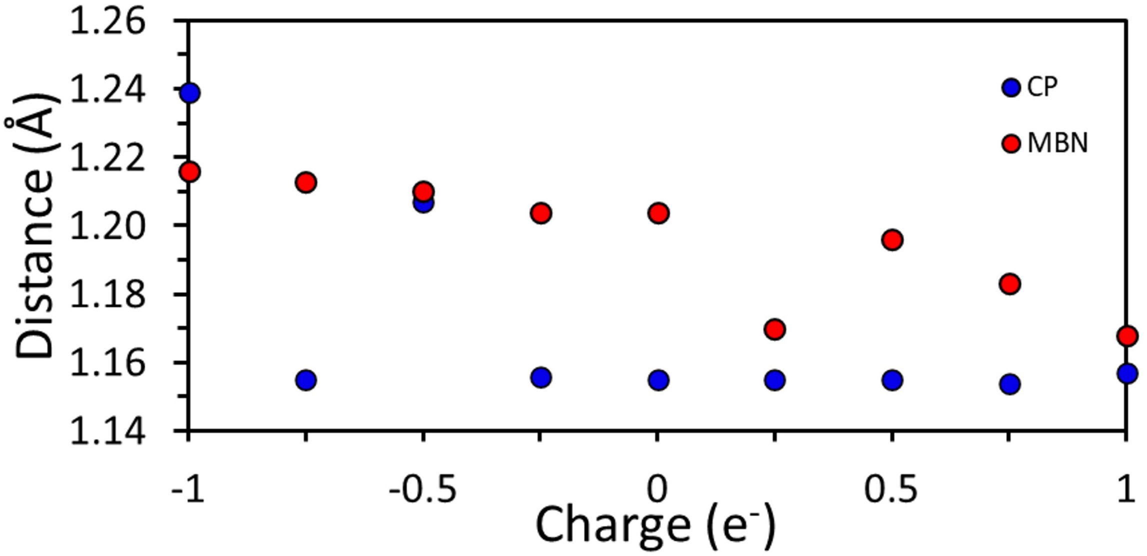

Figure 2: Calculated bond length of Au-O and Au-S for CP and MBN.

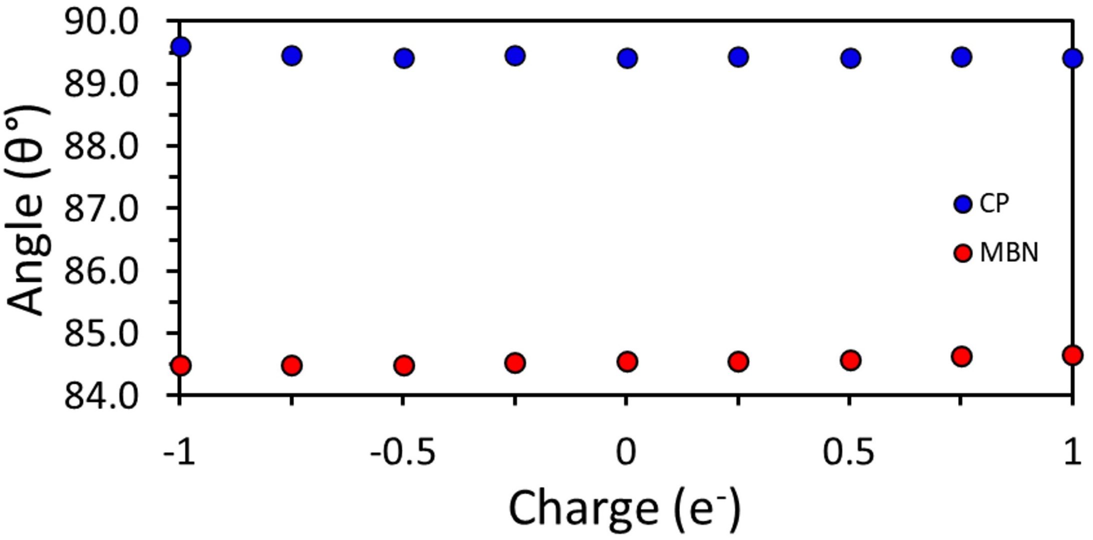

Figure 3: Calculated angle of the adsorbed molecule with respect to the surface normal for CP and MBN. The data suggest that MBN exhibits a slight tilt of a few degrees compared to CP.

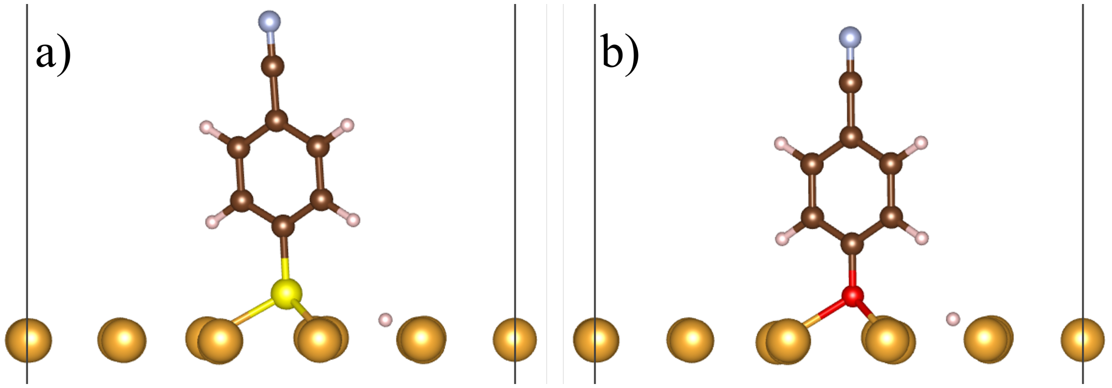

Figure 4: DFT-optimized structures of neutral a) 4-mercaptobenzonitirle (MBN) and b) 4-cyanophenols (CP). Optimized coordinates in VASP-POSCAR format are given below in the VASP input files section of the supporting information.

Input files

VASP input files: INCAR (Geometry Optimization) General characteristics

- System = $\text{Mol}_A u \quad CalculationTitlePREC = Accurate \quad Options : Normal—Single—Accurate$

- ENCUT = 520 Kinetic Energy(eV) Converge
- ISTART = 0 Job: 0-new 1-cont 2-samecut
- ICHARG = 2 Charge density: 1-file 2-atom 10-cons 11-DOS/BANDS
- ISPIN = 1 Spin Polarize: 1-No 2-Yes
- LWAVE = False Write the WAVECAR
- LCHARG = False Write the CHGCAR

Electronic Relaxation (SCF)

- NELM = 200 Max Number of Elec Self Cons Steps
- NELMIN = 4 Min Number of ESC steps
- NELMDL = 6 Number of non-SC at the beginning
- EDIFF = 1.0E-08 Stopping criteria for ESC
- LREAL = Auto Real space projection
- ALGO = Normal Electronic algorithm minimization: Normal | VeryFast | Fast | Conjugate

VDW Corrections

- IVDW = 21 11-D3, 12-D3$_B J, 20 - TS, 21 - TS/H$

Ionic Relaxation

- EDIFFG = -0.01   Stopping criteria for ionic self-cons steps
- NSW = 200   Max Number of ISC steps: 0-Single Point
- IBRION = 2   Ionic Relaxation Method: 0-MD   1-qNewton-RaphsonElectronic   2-CG
- ISIF = 2   Stress and Relaxation: 1-Single Point   2-Ion   3-Full
- SIGMA = 0.05   Insulators/semiconductors=0.1   metals=0.05
- ISMEAR = 0   Partial Occupancies for each Orbital: 0-Gaussian   -1-Fermi   -4 tetrahedron   -5 tetrahedron/Blöchl   -5 DOS, -2 from file, -1 Fermi Smear, 0 Gaussian Smear
- ISYM = 0   Parallelization
- NPAR = 8   cores per band sqrt num of cores: set to 8 if using 4 nodes with 16 cores each

Here is the LaTeX-compatible representation of the second VASP input file:

INCAR (Static Energy/Charge) General characteristics

- System = Mol_Au   Calculation Title
- PREC = Accurate   Options: Normal | Single | Accurate
- ENCUT = 520   Kinetic Energy(eV) Converge
- ISTART = 0   Job: 0-new   1-cont   2-samecut
- ICHARG = 2   Charge density: 1-file   2-atom   10-cons   11-DOS/BANDS
- ISPIN = 1   Spin Polarize: 1-No   2-Yes
- LWAVE = True   Write the WAVECAR

- LCHARG = True   Write the CHGCAR

Electronic Relaxation (SCF)

- NELM = 200   Max Number of Elec Self Cons Steps
- NELMIN = 4   Min Number of ESC steps
- NELMDL = 6   Number of non-SC at the beginning
- EDIFF = 1.0E-08   Stopping criteria for ESC
- LREAL = Auto   Real space projection
- ALGO = Normal   Electronic algorithm minimization: Normal | VeryFast | Fast | Conjugate

VDW Corrections

- IVDW = 21   11-D3, 12-D3_BJ, 20-TS, 21-TS/H

Ionic Relaxation

- EDIFFG = -0.01   Stopping criteria for ionic self-cons steps
- NSW = 0   Max Number of ISC steps: 0-Single Point
- IBRION = 2   Ionic Relaxation Method: 0-MD   1-qNewton-RaphsonElectronic   2-CG
- ISIF = 1   Stress and Relaxation: 1-Single Point   2-Ion   3-Full
- SIGMA = 0.05   Insulators/semiconductors=0.1   metals=0.05
- ISMEAR = 0   Partial Occupancies for each Orbital: 0-Gaussian   -1-Fermi   -4 tetrahedron   -5 tetrahedron/Blöchl   -5 DOS, -2 from file, -1 Fermi Smear, 0 Gaussian Smear

- ISYM = 0 Parallelization
- NPAR = 8 cores per band sqrt num of cores: set to 8 if using 4 nodes with 16 cores each

Here is the LaTeX-compatible representation of the third VASP input file:

INCAR (Frequency) General characteristics

- System = Mol_Au Calculation Title
- PREC = Accurate Options: Normal | Single | Accurate
- ENCUT = 520 Kinetic Energy(eV) Converge
- ISTART = 0 Job: 0-new 1-cont 2-samecut
- ICHARG = 2 Charge density: 1-file 2-atom 10-cons 11-DOS/BANDS
- ISPIN = 1 Spin Polarize: 1-No 2-Yes
- LWAVE = False Write the WAVECAR
- LCHARG = False Write the CHGCAR

Electronic Relaxation (SCF)

- NELM = 200 Max Number of Elec Self Cons Steps
- NELMIN = 4 Min Number of ESC steps
- NELMDL = 6 Number of non-SC at the beginning
- EDIFF = 1.0E-08 Stopping criteria for ESC
- LREAL = Auto Real space projection
- ALGO = Normal Electronic algorithm minimization: Normal | VeryFast | Fast | Conjugate

VDW Corrections

- IVDW = 21   11-D3, 12-D3_BJ, 20-TS, 21-TS/H

Ionic Relaxation

- EDIFFG = -0.01   Stopping criteria for ionic self-cons steps
- NSW = 200   Max Number of ISC steps: 0-Single Point
- IBRION = 5   Ionic Relaxation Method: 0-MD   1-qNewton-RaphsonElectronic   2-CG
- ISIF = 2   Stress and Relaxation: 1-Single Point   2-Ion   3-Full
- SIGMA = 0.05   Insulators/semiconductors=0.1   metals=0.05
- ISMEAR = 0   Partial Occupancies for each Orbital: 0-Gaussian   -1-Fermi   -4 tetrahedron   -5 tetrahedron/Blöchl   -5 DOS, -2 from file, -1 Fermi Smear, 0 Gaussian Smear
- ISYM = 0   Parallelization
- NPAR = 8   cores per band sqrt num of cores: set to 8 if using 4 nodes with 16 cores each

Here is the LaTeX-compatible representation of the KPOINTS section followed by the POTCAR-list:

KPOINTS

- Regular k-point mesh
- 0   ! 0 -¿ determine number of k points automatically
- Gamma   ! generate a Gamma centered mesh

- 1 1 1 ! subdivisions N_1, N_2 and N_3 along the reciprocal lattice vectors
- 0 0 0 ! optional shift of the mesh (s_1, s_2, s_3)

POTCAR-list

- Au: PAW_PBE Au 04Oct2007
- N: PAW_PBE N 08Apr2002
- C: PAW_PBE C 08Apr2002
- H: PAW_PBE H 15Jun2001
- O: PAW_PBE O 08Apr2002
- S: PAW_PBE S 06Sep2000

Geometry Optimization for Neutral MBN

```
POSCAR (Optimized Geometry Neutral: MBZ)


Sulfur
   1.00000000000000
    14.7477998734000000     0.0000000000000000     0.0000000000000000
    -7.3738999367000000    12.7719693403000001     0.0000000000000000
     0.0000000000000000     0.0000000000000000    39.6332015990999977
   Au   S    N    C    H
   125     1     1     7     5
Selective dynamics
Direct
  0.1333300020000010  0.0666700009999985  0.0000000000000000   F   F   F
```

0.3333300050000005	0.0666700009999985	0.0000000000000000	F	F	F
0.5333300229999978	0.0666700009999985	0.0000000000000000	F	F	F
0.7333300109999996	0.0666700009999985	0.0000000000000000	F	F	F
0.9333299990000015	0.0666700009999985	0.0000000000000000	F	F	F
0.1333300020000010	0.2666699890000004	0.0000000000000000	F	F	F
0.3333300050000005	0.2666699890000004	0.0000000000000000	F	F	F
0.5333300229999978	0.2666699890000004	0.0000000000000000	F	F	F
0.7333300109999996	0.2666699890000004	0.0000000000000000	F	F	F
0.9333299990000015	0.2666699890000004	0.0000000000000000	F	F	F
0.1333300020000010	0.4666700069999976	0.0000000000000000	F	F	F
0.3333300050000005	0.4666700069999976	0.0000000000000000	F	F	F
0.5333300229999978	0.4666700069999976	0.0000000000000000	F	F	F
0.7333300109999996	0.4666700069999976	0.0000000000000000	F	F	F
0.9333299990000015	0.4666700069999976	0.0000000000000000	F	F	F
0.1333300020000010	0.6666700239999983	0.0000000000000000	F	F	F
0.3333300050000005	0.6666700239999983	0.0000000000000000	F	F	F
0.5333300229999978	0.6666700239999983	0.0000000000000000	F	F	F
0.7333300109999996	0.6666700239999983	0.0000000000000000	F	F	F
0.9333299990000015	0.6666700239999983	0.0000000000000000	F	F	F
0.1333300020000010	0.8666700120000002	0.0000000000000000	F	F	F
0.3333300050000005	0.8666700120000002	0.0000000000000000	F	F	F
0.5333300229999978	0.8666700120000002	0.0000000000000000	F	F	F
0.7333300109999996	0.8666700120000002	0.0000000000000000	F	F	F
0.9333299990000015	0.8666700120000002	0.0000000000000000	F	F	F
0.0000000000000000	0.0000000000000000	0.0607599989999983	F	F	F
0.2000000029999995	0.0000000000000000	0.0607599989999983	F	F	F
0.4000000059999991	0.0000000000000000	0.0607599989999983	F	F	F

0.6000000240000034	0.0000000000000000	0.0607599989999983	F	F	F
0.8000000119999982	0.0000000000000000	0.0607599989999983	F	F	F
0.0000000000000000	0.2000000029999995	0.0607599989999983	F	F	F
0.2000000029999995	0.2000000029999995	0.0607599989999983	F	F	F
0.4000000059999991	0.2000000029999995	0.0607599989999983	F	F	F
0.6000000240000034	0.2000000029999995	0.0607599989999983	F	F	F
0.8000000119999982	0.2000000029999995	0.0607599989999983	F	F	F
0.0000000000000000	0.4000000059999991	0.0607599989999983	F	F	F
0.2000000029999995	0.4000000059999991	0.0607599989999983	F	F	F
0.4000000059999991	0.4000000059999991	0.0607599989999983	F	F	F
0.6000000240000034	0.4000000059999991	0.0607599989999983	F	F	F
0.8000000119999982	0.4000000059999991	0.0607599989999983	F	F	F
0.0000000000000000	0.6000000240000034	0.0607599989999983	F	F	F
0.2000000029999995	0.6000000240000034	0.0607599989999983	F	F	F
0.4000000059999991	0.6000000240000034	0.0607599989999983	F	F	F
0.6000000240000034	0.6000000240000034	0.0607599989999983	F	F	F
0.8000000119999982	0.6000000240000034	0.0607599989999983	F	F	F
0.0000000000000000	0.8000000119999982	0.0607599989999983	F	F	F
0.2000000029999995	0.8000000119999982	0.0607599989999983	F	F	F
0.4000000059999991	0.8000000119999982	0.0607599989999983	F	F	F
0.6000000240000034	0.8000000119999982	0.0607599989999983	F	F	F
0.8000000119999982	0.8000000119999982	0.0607599989999983	F	F	F
0.0669353353772593	0.1336458211244600	0.1191733915068130	T	T	T
0.2678214884495306	0.1356163187020301	0.1193292499754469	T	T	T
0.4667297147376390	0.1333388749501563	0.1192967534601989	T	T	T
0.6667737581269840	0.1331084514613844	0.1193228030047090	T	T	T
0.8669882059954841	0.1330115413533221	0.1192186799407149	T	T	T

0.0678119453239899	0.3344390998359034	0.1196008025531616	T	T	T
0.2681977761278779	0.3354263639851447	0.1198640935297388	T	T	T
0.4671682093761588	0.3354024412620332	0.1197822975209635	T	T	T
0.6664733739233059	0.3334661550030890	0.1193384347261924	T	T	T
0.8668940952801780	0.3332195507180009	0.1193186835996515	T	T	T
0.0670958500247900	0.5330542318480859	0.1192448980290330	T	T	T
0.2678400177512925	0.5325853741029851	0.1196710808925665	T	T	T
0.4664562110339032	0.5335753782646734	0.1204044756697260	T	T	T
0.6645387444647968	0.5327422428793611	0.1198166373653168	T	T	T
0.8666302294797664	0.5332033561299162	0.1192903607328607	T	T	T
0.0671456149860482	0.7336916108888766	0.1188580385835852	T	T	T
0.2678398979925591	0.7323154796129344	0.1192196018002585	T	T	T
0.4677458022093937	0.7322896633758519	0.1196934892445522	T	T	T
0.6647279193363447	0.7317030661607371	0.1198877599175233	T	T	T
0.8642405490292308	0.7319502089267049	0.1193759827274116	T	T	T
0.0673584070835845	0.9324774879847836	0.1190132244695909	T	T	T
0.2663152296097704	0.9328668452881160	0.1188624272514968	T	T	T
0.4670659611002558	0.9329191408324913	0.1192424328027806	T	T	T
0.6657284009423062	0.9321835854099648	0.1196102241733965	T	T	T
0.8664004338335735	0.9330051248607345	0.1191798112667330	T	T	T
0.1321739735898414	0.0641159932181198	0.1773093298818423	T	T	T
0.3318564565788901	0.0638598519829916	0.1773040611985132	T	T	T
0.5340051388670385	0.0664006885095486	0.1779000355119799	T	T	T
0.7341447398771231	0.0665308149996433	0.1778647776675685	T	T	T
0.9335031079515980	0.0663929434224365	0.1780213561247072	T	T	T
0.1331257901500348	0.2650539221090906	0.1775793129916735	T	T	T
0.3318481874270311	0.2643412371144639	0.1783151082580958	T	T	T

0.5319119145860363	0.2653140164489816	0.1774668576126510	T	T	T
0.7336457855343049	0.2663524120746357	0.1778448760623599	T	T	T
0.9334716342190099	0.2658815929037179	0.1778617641019536	T	T	T
0.1343088969838253	0.4693235715341788	0.1782128017572182	T	T	T
0.3348041156633814	0.4682719152028633	0.1808304005420674	T	T	T
0.5315943089014938	0.4680188393079062	0.1807302578818792	T	T	T
0.7347747177378720	0.4679888653232291	0.1774748876645999	T	T	T
0.9337206149859799	0.4660312543575458	0.1778898153278690	T	T	T
0.1334778314910220	0.6675619036185376	0.1776366500736313	T	T	T
0.3323253297993890	0.6682294094928488	0.1781100791207115	T	T	T
0.5324498321207324	0.6651191795606604	0.1808864780376911	T	T	T
0.7354871669546725	0.6676185275285634	0.1784673662266082	T	T	T
0.9364146012574773	0.6680995622017477	0.1773064872983210	T	T	T
0.1346314875876400	0.8654319530513149	0.1784127195229744	T	T	T
0.3326607709599075	0.8669443066858729	0.1776305550431033	T	T	T
0.5309540494581709	0.8658665235532536	0.1782305600638395	T	T	T
0.7349973238409128	0.8666953231923348	0.1776155669496055	T	T	T
0.9356592795084273	0.8674990283369564	0.1773326149751214	T	T	T
0.9993699104105402	0.0001711256323784	0.2390857064489470	T	T	T
0.1985257454688386	0.9984902322326717	0.2386207715106717	T	T	T
0.6045432002275863	0.0053345835519449	0.2388817969610063	T	T	T
0.4022727268429631	0.0027176791355175	0.2383171234216017	T	T	T
0.1887658344848526	0.1892585744189454	0.2383008513485959	T	T	T
0.9960647541126328	0.1975007608895728	0.2392151096284724	T	T	T
0.8022055144835746	0.0035828091915124	0.2392055252006854	T	T	T
0.1890626281680770	0.3933317117581067	0.2367120697802921	T	T	T
0.3837379474505210	0.3702121861727246	0.2391391914753458	T	T	T

0.6049384783779932	0.3952121581107075	0.2360646869980823	T	T	T
0.8002011684721329	0.1997679649389697	0.2388683566552892	T	T	T
0.9948320737140663	0.3954547659265130	0.2388743436109167	T	T	T
0.6024651648120073	0.1991868963486247	0.2385812171609392	T	T	T
0.3997765013767615	0.1903203866619961	0.2379417342053956	T	T	T
0.8010769726380936	0.3975473582343981	0.2385614013242178	T	T	T
0.9981340854721862	0.5978163117487971	0.2382849945859356	T	T	T
0.3860431967194342	0.6158216375673438	0.2386749631641688	T	T	T
0.1855106757724194	0.5924704168964174	0.2403215299394603	T	T	T
0.0017571286020636	0.8016592670224764	0.2386273734726210	T	T	T
0.8106353509532740	0.6003208453606176	0.2379775511048860	T	T	T
0.6301895202420029	0.6149052964776651	0.2395343204750301	T	T	T
0.1970844218386532	0.8031121768811136	0.2405688358611665	T	T	T
0.4073663893874037	0.8153397402947123	0.2403114628011592	T	T	T
0.8103061670235209	0.8106192125371238	0.2383369630144409	T	T	T
0.6065350261872920	0.8107399015237395	0.2367434460809688	T	T	T
0.4674050297468147	0.5321774987060086	0.2707456855126424	T	T	T
0.4892574605134009	0.4947710758333475	0.4501823008560624	T	T	T
0.4718056509902190	0.5243790419843228	0.3152103222379374	T	T	T
0.3899499195657559	0.4392791892344678	0.3321533018597861	T	T	T
0.5576249919067974	0.6024361466896196	0.3329880996827564	T	T	T
0.3942142962198384	0.4320522131549643	0.3670256261121457	T	T	T
0.5618153750289239	0.5951193216435525	0.3678716880306210	T	T	T
0.4802922568829550	0.5097542666329562	0.3851362447791363	T	T	T
0.4851814471156546	0.5016203035909210	0.4209053103127460	T	T	T
0.2657733470992736	0.7339336845235152	0.2563485047475945	T	T	T
0.3241256849993786	0.3788955284653956	0.3178788139447954	T	T	T

```
  0.6209360109009079  0.6676325158823393  0.3193531666958053   T   T   T
  0.3314512428238639  0.3660637585952100  0.3804845405544966   T   T   T
  0.6284168136516245  0.6548610209458000  0.3819621244584321   T   T   T
```

Geometry Optimization Neutral for CP

```
POSCAR (Optimized Geometry Neutral: CP)
Oxygen
   1.00000000000000
    14.7477998734000000    0.0000000000000000    0.0000000000000000
    -7.3738999367000000   12.7719693403000001    0.0000000000000000
     0.0000000000000000    0.0000000000000000   39.6332015990999977
   Au   O    N    C    H
   125    1    1    7    5
Selective dynamics
Direct
  0.1333300020000010  0.0666700009999985  0.0000000000000000   F   F   F
  0.3333300050000005  0.0666700009999985  0.0000000000000000   F   F   F
  0.5333300229999978  0.0666700009999985  0.0000000000000000   F   F   F
  0.7333300109999996  0.0666700009999985  0.0000000000000000   F   F   F
  0.9333299990000015  0.0666700009999985  0.0000000000000000   F   F   F
```

0.1333300020000010	0.2666699890000004	0.0000000000000000	F	F	F
0.3333300050000005	0.2666699890000004	0.0000000000000000	F	F	F
0.5333300229999978	0.2666699890000004	0.0000000000000000	F	F	F
0.7333300109999996	0.2666699890000004	0.0000000000000000	F	F	F
0.9333299990000015	0.2666699890000004	0.0000000000000000	F	F	F
0.1333300020000010	0.4666700069999976	0.0000000000000000	F	F	F
0.3333300050000005	0.4666700069999976	0.0000000000000000	F	F	F
0.5333300229999978	0.4666700069999976	0.0000000000000000	F	F	F
0.7333300109999996	0.4666700069999976	0.0000000000000000	F	F	F
0.9333299990000015	0.4666700069999976	0.0000000000000000	F	F	F
0.1333300020000010	0.6666700239999983	0.0000000000000000	F	F	F
0.3333300050000005	0.6666700239999983	0.0000000000000000	F	F	F
0.5333300229999978	0.6666700239999983	0.0000000000000000	F	F	F
0.7333300109999996	0.6666700239999983	0.0000000000000000	F	F	F
0.9333299990000015	0.6666700239999983	0.0000000000000000	F	F	F
0.1333300020000010	0.8666700120000002	0.0000000000000000	F	F	F
0.3333300050000005	0.8666700120000002	0.0000000000000000	F	F	F
0.5333300229999978	0.8666700120000002	0.0000000000000000	F	F	F
0.7333300109999996	0.8666700120000002	0.0000000000000000	F	F	F
0.9333299990000015	0.8666700120000002	0.0000000000000000	F	F	F
0.0000000000000000	0.0000000000000000	0.0607599989999983	F	F	F
0.2000000029999995	0.0000000000000000	0.0607599989999983	F	F	F
0.4000000059999991	0.0000000000000000	0.0607599989999983	F	F	F
0.6000000240000034	0.0000000000000000	0.0607599989999983	F	F	F
0.8000000119999982	0.0000000000000000	0.0607599989999983	F	F	F
0.0000000000000000	0.2000000029999995	0.0607599989999983	F	F	F
0.2000000029999995	0.2000000029999995	0.0607599989999983	F	F	F

0.4000000059999991	0.2000000029999995	0.0607599989999983	F	F	F
0.6000000240000034	0.2000000029999995	0.0607599989999983	F	F	F
0.8000000119999982	0.2000000029999995	0.0607599989999983	F	F	F
0.0000000000000000	0.4000000059999991	0.0607599989999983	F	F	F
0.2000000029999995	0.4000000059999991	0.0607599989999983	F	F	F
0.4000000059999991	0.4000000059999991	0.0607599989999983	F	F	F
0.6000000240000034	0.4000000059999991	0.0607599989999983	F	F	F
0.8000000119999982	0.4000000059999991	0.0607599989999983	F	F	F
0.0000000000000000	0.6000000240000034	0.0607599989999983	F	F	F
0.2000000029999995	0.6000000240000034	0.0607599989999983	F	F	F
0.4000000059999991	0.6000000240000034	0.0607599989999983	F	F	F
0.6000000240000034	0.6000000240000034	0.0607599989999983	F	F	F
0.8000000119999982	0.6000000240000034	0.0607599989999983	F	F	F
0.0000000000000000	0.8000000119999982	0.0607599989999983	F	F	F
0.2000000029999995	0.8000000119999982	0.0607599989999983	F	F	F
0.4000000059999991	0.8000000119999982	0.0607599989999983	F	F	F
0.6000000240000034	0.8000000119999982	0.0607599989999983	F	F	F
0.8000000119999982	0.8000000119999982	0.0607599989999983	F	F	F
0.0668419722876899	0.1333519375879540	0.1192430153116466	T	T	T
0.2674986667624878	0.1345637933690094	0.1193067131782227	T	T	T
0.4667925278251343	0.1330556318266397	0.1193672197351545	T	T	T
0.6669200689538085	0.1331223770228195	0.1193899581554902	T	T	T
0.8669837764042310	0.1329980769929051	0.1192715446805889	T	T	T
0.0675335991067439	0.3341307122769073	0.1196369962584811	T	T	T
0.2677216782423930	0.3346935625933163	0.1197788090643982	T	T	T
0.4673652553088601	0.3348539955530865	0.1197434770834948	T	T	T
0.6667395746636879	0.3333007560992612	0.1193930524548890	T	T	T

0.8668846675500650	0.3330882174136878	0.1193891006084128	T	T	T
0.0669595823168315	0.5330003813274703	0.1193010808670184	T	T	T
0.2672319017377334	0.5322304579087528	0.1195632136287742	T	T	T
0.4666044420409141	0.5334109429158428	0.1201436891037809	T	T	T
0.6651667922376788	0.5326669761142268	0.1197451358228363	T	T	T
0.8669789489533517	0.5332310090739248	0.1193663620233502	T	T	T
0.0670362552837932	0.7335948034618452	0.1190516431648709	T	T	T
0.2670916497225458	0.7329173600909398	0.1190689531001839	T	T	T
0.4677640463144510	0.7327483423131557	0.1195700997257981	T	T	T
0.6652768479172247	0.7322540062606679	0.1197891152353857	T	T	T
0.8653881440230996	0.7324847074333962	0.1193162867759815	T	T	T
0.0673968891218248	0.9325877648586574	0.1192214250547884	T	T	T
0.2664302808630410	0.9329741503137463	0.1190548965329388	T	T	T
0.4670101570479469	0.9330503728315047	0.1193005784428507	T	T	T
0.6658500490328239	0.9324426019253446	0.1196355811865105	T	T	T
0.8666297810811555	0.9331432810081880	0.1192422899369091	T	T	T
0.1322420606689438	0.0644316180712475	0.1776061396291171	T	T	T
0.3323843847325296	0.0640356498021562	0.1776211676406885	T	T	T
0.5341501949404072	0.0662768143368201	0.1780282084069133	T	T	T
0.7343194151065637	0.0665105114616721	0.1779839720393613	T	T	T
0.9336426925072725	0.0663709205596037	0.1781640794631055	T	T	T
0.1329228545746830	0.2651339146027638	0.1777573567644033	T	T	T
0.3314398981720555	0.2626183272239438	0.1778306129514799	T	T	T
0.5326400396330602	0.2651775658839238	0.1776433914909578	T	T	T
0.7338766590741838	0.2661375818928889	0.1780063461232167	T	T	T
0.9334796378160822	0.2656593162320042	0.1779841003589943	T	T	T
0.1339800876313002	0.4686276253172661	0.1783802081323531	T	T	T

0.3344636766365572	0.4675702107530204	0.1798853006528664	T	T	T
0.5324117802389401	0.4677341172404394	0.1799990627063817	T	T	T
0.7348864826398076	0.4674214095234068	0.1776445039630168	T	T	T
0.9337359957182556	0.4658539608449587	0.1780277611050924	T	T	T
0.1330062131057347	0.6669562953926284	0.1778840401131123	T	T	T
0.3311892866822903	0.6687834933996111	0.1773240585790783	T	T	T
0.5324301630746673	0.6655373324962661	0.1799179789966009	T	T	T
0.7373571085127993	0.6685848453564243	0.1778740819033674	T	T	T
0.9359945958339788	0.6676778869052316	0.1776192627996717	T	T	T
0.1346873222666589	0.8653668045981588	0.1785712498941527	T	T	T
0.3330605131014151	0.8669951599284240	0.1778854859733479	T	T	T
0.5313480778899830	0.8660004566373561	0.1783825736255601	T	T	T
0.7348559205770420	0.8670668508055537	0.1777585448662104	T	T	T
0.9356054726227963	0.8677932471726969	0.1776064835629910	T	T	T
0.9999821941283772	0.0001969767598283	0.2391920286687918	T	T	T
0.1991154060742036	0.9989617531926015	0.2385902784535267	T	T	T
0.6042287278851283	0.0037706340449630	0.2387435392869113	T	T	T
0.4022743915010868	0.0020283572820636	0.2383300423955782	T	T	T
0.1913906970232577	0.1916352334376257	0.2385340760175489	T	T	T
0.9975080947326447	0.1981615889534070	0.2391459226211610	T	T	T
0.8018977880923139	0.0025866370786401	0.2391320380131507	T	T	T
0.1933533320818877	0.3951859334578200	0.2371086544095017	T	T	T
0.3872912434640545	0.3760255577977731	0.2369268800289292	T	T	T
0.6035350183178908	0.3965705895745860	0.2364412714172444	T	T	T
0.8004801962318773	0.1995390163558421	0.2389728761856164	T	T	T
0.9962433858288995	0.3957536575097572	0.2387494230264397	T	T	T
0.6024072438757899	0.1993161885680031	0.2385854136119616	T	T	T

0.4005332409075817	0.1923734682121555	0.2382592276370034	T	T	T
0.8007257149766984	0.3976192758803189	0.2385886100721764	T	T	T
0.9980247330445184	0.5977536712531227	0.2383297093062172	T	T	T
0.3885703183767685	0.6113259670247191	0.2364020322760943	T	T	T
0.1881061112224378	0.5923105779777933	0.2405198942773482	T	T	T
0.0011182852755258	0.8009622037889623	0.2385894533164796	T	T	T
0.8077285107020685	0.5995336007542822	0.2382605589374500	T	T	T
0.6242106190856462	0.6129849485084148	0.2370279069865333	T	T	T
0.1975626335152011	0.8024722662760131	0.2402994990129836	T	T	T
0.4076638259511939	0.8118575427192846	0.2405115217263928	T	T	T
0.8085553791422918	0.8088216032256096	0.2385342167887806	T	T	T
0.6048389896539236	0.8067241944156246	0.2371165938957071	T	T	T
0.4711741691691134	0.5293104958651131	0.2700386923140111	T	T	T
0.4756371642897003	0.5168395804299210	0.4405963345174230	T	T	T
0.4715731475187446	0.5277037431657124	0.3043011161417351	T	T	T
0.3875252832517814	0.4462913177037426	0.3219896158162970	T	T	T
0.5559173476755517	0.6072464120147499	0.3224202109853910	T	T	T
0.3882923036426023	0.4438165944718118	0.3569934777464760	T	T	T
0.5568047373054421	0.6049073642371250	0.3574349227671350	T	T	T
0.4731241131909423	0.5229699297824547	0.3751731361936348	T	T	T
0.4744630975107086	0.5197087869679374	0.4111131514956117	T	T	T
0.2665945246755480	0.7333847931390541	0.2565898474903253	T	T	T
0.3223053968995510	0.3850284629597612	0.3076848448490287	T	T	T
0.6206210600723956	0.6702296790092115	0.3084593585480721	T	T	T
0.3234553193601448	0.3802920467042860	0.3706220118224955	T	T	T
0.6225668691219184	0.6662399661308390	0.3714061160443090	T	T	T

POSCAR (Optimized Geometry Neutral: CP)

Oxygen

```
   1.00000000000000
    14.7477998734000000    0.0000000000000000    0.0000000000000000
    -7.3738999367000000   12.7719693403000001    0.0000000000000000
     0.0000000000000000    0.0000000000000000   39.6332015990999977
   Au    O    N    C    H
   125     1     1     7     5
Selective dynamics
Direct
  0.1333300020000010  0.0666700009999985  0.0000000000000000   F   F   F
  0.3333300050000005  0.0666700009999985  0.0000000000000000   F   F   F
  0.5333300229999978  0.0666700009999985  0.0000000000000000   F   F   F
  0.7333300109999996  0.0666700009999985  0.0000000000000000   F   F   F
  0.9333299990000015  0.0666700009999985  0.0000000000000000   F   F   F
  0.1333300020000010  0.2666699890000004  0.0000000000000000   F   F   F
  0.3333300050000005  0.2666699890000004  0.0000000000000000   F   F   F
  0.5333300229999978  0.2666699890000004  0.0000000000000000   F   F   F
  0.7333300109999996  0.2666699890000004  0.0000000000000000   F   F   F
  0.9333299990000015  0.2666699890000004  0.0000000000000000   F   F   F
  0.1333300020000010  0.4666700069999976  0.0000000000000000   F   F   F
  0.3333300050000005  0.4666700069999976  0.0000000000000000   F   F   F
  0.5333300229999978  0.4666700069999976  0.0000000000000000   F   F   F
  0.7333300109999996  0.4666700069999976  0.0000000000000000   F   F   F
  0.9333299990000015  0.4666700069999976  0.0000000000000000   F   F   F
  0.1333300020000010  0.6666700239999983  0.0000000000000000   F   F   F
```

0.3333300050000005	0.6666700239999983	0.0000000000000000	F	F	F
0.5333300229999978	0.6666700239999983	0.0000000000000000	F	F	F
0.7333300109999996	0.6666700239999983	0.0000000000000000	F	F	F
0.9333299990000015	0.6666700239999983	0.0000000000000000	F	F	F
0.1333300020000010	0.8666700120000002	0.0000000000000000	F	F	F
0.3333300050000005	0.8666700120000002	0.0000000000000000	F	F	F
0.5333300229999978	0.8666700120000002	0.0000000000000000	F	F	F
0.7333300109999996	0.8666700120000002	0.0000000000000000	F	F	F
0.9333299990000015	0.8666700120000002	0.0000000000000000	F	F	F
0.0000000000000000	0.0000000000000000	0.0607599989999983	F	F	F
0.2000000029999995	0.0000000000000000	0.0607599989999983	F	F	F
0.4000000059999991	0.0000000000000000	0.0607599989999983	F	F	F
0.6000000240000034	0.0000000000000000	0.0607599989999983	F	F	F
0.8000000119999982	0.0000000000000000	0.0607599989999983	F	F	F
0.0000000000000000	0.2000000029999995	0.0607599989999983	F	F	F
0.2000000029999995	0.2000000029999995	0.0607599989999983	F	F	F
0.4000000059999991	0.2000000029999995	0.0607599989999983	F	F	F
0.6000000240000034	0.2000000029999995	0.0607599989999983	F	F	F
0.8000000119999982	0.2000000029999995	0.0607599989999983	F	F	F
0.0000000000000000	0.4000000059999991	0.0607599989999983	F	F	F
0.2000000029999995	0.4000000059999991	0.0607599989999983	F	F	F
0.4000000059999991	0.4000000059999991	0.0607599989999983	F	F	F
0.6000000240000034	0.4000000059999991	0.0607599989999983	F	F	F
0.8000000119999982	0.4000000059999991	0.0607599989999983	F	F	F
0.0000000000000000	0.6000000240000034	0.0607599989999983	F	F	F
0.2000000029999995	0.6000000240000034	0.0607599989999983	F	F	F
0.4000000059999991	0.6000000240000034	0.0607599989999983	F	F	F

0.6000000240000034	0.6000000240000034	0.0607599989999983	F	F	F
0.8000000119999982	0.6000000240000034	0.0607599989999983	F	F	F
0.0000000000000000	0.8000000119999982	0.0607599989999983	F	F	F
0.2000000029999995	0.8000000119999982	0.0607599989999983	F	F	F
0.4000000059999991	0.8000000119999982	0.0607599989999983	F	F	F
0.6000000240000034	0.8000000119999982	0.0607599989999983	F	F	F
0.8000000119999982	0.8000000119999982	0.0607599989999983	F	F	F
0.0668419722876899	0.1333519375879540	0.1192430153116466	T	T	T
0.2674986667624878	0.1345637933690094	0.1193067131782227	T	T	T
0.4667925278251343	0.1330556318266397	0.1193672197351545	T	T	T
0.6669200689538085	0.1331223770228195	0.1193899581554902	T	T	T
0.8669837764042310	0.1329980769929051	0.1192715446805889	T	T	T
0.0675335991067439	0.3341307122769073	0.1196369962584811	T	T	T
0.2677216782423930	0.3346935625933163	0.1197788090643982	T	T	T
0.4673652553088601	0.3348539955530865	0.1197434770834948	T	T	T
0.6667395746636879	0.3333007560992612	0.1193930524548890	T	T	T
0.8668846675500650	0.3330882174136878	0.1193891006084128	T	T	T
0.0669595823168315	0.5330003813274703	0.1193010808670184	T	T	T
0.2672319017377334	0.5322304579087528	0.1195632136287742	T	T	T
0.4666044420409141	0.5334109429158428	0.1201436891037809	T	T	T
0.6651667922376788	0.5326669761142268	0.1197451358228363	T	T	T
0.8669789489533517	0.5332310090739248	0.1193663620233502	T	T	T
0.0670362552837932	0.7335948034618452	0.1190516431648709	T	T	T
0.2670916497225458	0.7329173600909398	0.1190689531001839	T	T	T
0.4677640463144510	0.7327483423131557	0.1195700997257981	T	T	T
0.6652768479172247	0.7322540062606679	0.1197891152353857	T	T	T
0.8653881440230996	0.7324847074333962	0.1193162867759815	T	T	T

0.0673968891218248	0.9325877648586574	0.1192214250547884	T	T	T
0.2664302808630410	0.9329741503137463	0.1190548965329388	T	T	T
0.4670101570479469	0.9330503728315047	0.1193005784428507	T	T	T
0.6658500490328239	0.9324426019253446	0.1196355811865105	T	T	T
0.8666297810811555	0.9331432810081880	0.1192422899369091	T	T	T
0.1322420606689438	0.0644316180712475	0.1776061396291171	T	T	T
0.3323843847325296	0.0640356498021562	0.1776211676406885	T	T	T
0.5341501949404072	0.0662768143368201	0.1780282084069133	T	T	T
0.7343194151065637	0.0665105114616721	0.1779839720393613	T	T	T
0.9336426925072725	0.0663709205596037	0.1781640794631055	T	T	T
0.1329228545746830	0.2651339146027638	0.1777573567644033	T	T	T
0.3314398981720555	0.2626183272239438	0.1778306129514799	T	T	T
0.5326400396330602	0.2651775658839238	0.1776433914909578	T	T	T
0.7338766590741838	0.2661375818928889	0.1780063461232167	T	T	T
0.9334796378160822	0.2656593162320042	0.1779841003589943	T	T	T
0.1339800876313002	0.4686276253172661	0.1783802081323531	T	T	T
0.3344636766365572	0.4675702107530204	0.1798853006528664	T	T	T
0.5324117802389401	0.4677341172404394	0.1799990627063817	T	T	T
0.7348864826398076	0.4674214095234068	0.1776445039630168	T	T	T
0.9337359957182556	0.4658539608449587	0.1780277611050924	T	T	T
0.1330062131057347	0.6669562953926284	0.1778840401131123	T	T	T
0.3311892866822903	0.6687834933996111	0.1773240585790783	T	T	T
0.5324301630746673	0.6655373324962661	0.1799179789966009	T	T	T
0.7373571085127993	0.6685848453564243	0.1778740819033674	T	T	T
0.9359945958339788	0.6676778869052316	0.1776192627996717	T	T	T
0.1346873222666589	0.8653668045981588	0.1785712498941527	T	T	T
0.3330605131014151	0.8669951599284240	0.1778854859733479	T	T	T

0.5313480778899830	0.8660004566373561	0.1783825736255601	T	T	T
0.7348559205770420	0.8670668508055537	0.1777585448662104	T	T	T
0.9356054726227963	0.8677932471726969	0.1776064835629910	T	T	T
0.9999821941283772	0.0001969767598283	0.2391920286687918	T	T	T
0.1991154060742036	0.9989617531926015	0.2385902784535267	T	T	T
0.6042287278851283	0.0037706340449630	0.2387435392869113	T	T	T
0.4022743915010868	0.0020283572820636	0.2383300423955782	T	T	T
0.1913906970232577	0.1916352334376257	0.2385340760175489	T	T	T
0.9975080947326447	0.1981615889534070	0.2391459226211610	T	T	T
0.8018977880923139	0.0025866370786401	0.2391320380131507	T	T	T
0.1933533320818877	0.3951859334578200	0.2371086544095017	T	T	T
0.3872912434640545	0.3760255577977731	0.2369268800289292	T	T	T
0.6035350183178908	0.3965705895745860	0.2364412714172444	T	T	T
0.8004801962318773	0.1995390163558421	0.2389728761856164	T	T	T
0.9962433858288995	0.3957536575097572	0.2387494230264397	T	T	T
0.6024072438757899	0.1993161885680031	0.2385854136119616	T	T	T
0.4005332409075817	0.1923734682121555	0.2382592276370034	T	T	T
0.8007257149766984	0.3976192758803189	0.2385886100721764	T	T	T
0.9980247330445184	0.5977536712531227	0.2383297093062172	T	T	T
0.3885703183767685	0.6113259670247191	0.2364020322760943	T	T	T
0.1881061112224378	0.5923105779777933	0.2405198942773482	T	T	T
0.0011182852755258	0.8009622037889623	0.2385894533164796	T	T	T
0.8077285107020685	0.5995336007542822	0.2382605589374500	T	T	T
0.6242106190856462	0.6129849485084148	0.2370279069865333	T	T	T
0.1975626335152011	0.8024722662760131	0.2402994990129836	T	T	T
0.4076638259511939	0.8118575427192846	0.2405115217263928	T	T	T
0.8085553791422918	0.8088216032256096	0.2385342167887806	T	T	T

0.6048389896539236	0.8067241944156246	0.2371165938957071	T	T	T
0.4711741691691134	0.5293104958651131	0.2700386923140111	T	T	T
0.4756371642897003	0.5168395804299210	0.4405963345174230	T	T	T
0.4715731475187446	0.5277037431657124	0.3043011161417351	T	T	T
0.3875252832517814	0.4462913177037426	0.3219896158162970	T	T	T
0.5559173476755517	0.6072464120147499	0.3224202109853910	T	T	T
0.3882923036426023	0.4438165944718118	0.3569934777464760	T	T	T
0.5568047373054421	0.6049073642371250	0.3574349227671350	T	T	T
0.4731241131909423	0.5229699297824547	0.3751731361936348	T	T	T
0.4744630975107086	0.5197087869679374	0.4111131514956117	T	T	T
0.2665945246755480	0.7333847931390541	0.2565898474903253	T	T	T
0.3223053968995510	0.3850284629597612	0.3076848448490287	T	T	T
0.6206210600723956	0.6702296790092115	0.3084593585480721	T	T	T
0.3234553193601448	0.3802920467042860	0.3706220118224955	T	T	T
0.6225668691219184	0.6662399661308390	0.3714061160443090	T	T	T
